# Clinical and Non-Clinical Complaints Towards a Mental Health Service in the West of Ireland Over a Seven-Year Period

**DOI:** 10.1192/bjo.2024.501

**Published:** 2024-08-01

**Authors:** Andrew O'Malley, Elaine Walsh

**Affiliations:** 1 Department of Psychiatry, Galway University Hospital, Galway, Co. Galway, Ireland; 2 Department of Psychiatry of Old Age, Mayo University Hospital, Castlebar, Co. Mayo, Ireland

## Abstract

**Aims:**

To examine whether the rate of clinical and non-clinical complaints towards a mental health service (MHS) in the west of Ireland has changed over the preceding seven years. We aim to clarify the pathways for managing clinical and non-clinical complaints locally and compare with other MHS nationally. We aim to capture the nature of complaints, potential factors in any change in rate and quantify associated workloads via survey of senior clinicians involved in managing complaints.

**Methods:**

We obtained anonymous data from a local database maintained by administrative staff regarding annual complaint numbers for the previous seven years (2016–2022). Data separating clinical and non-clinical complaints were available for the previous four years only due to previous recording practices. Current complaint pathways were captured via administrative staff. A survey via telephone or email of Executive Clinical Directors (ECDs) typically involved in complaint management was conducted.

**Results:**

Annual rates of complaints have increased in the past four years, with these representing higher totals than any of the three previous years (2019–2022, n = 27,23,23,46 v. 2016–2018, n = 21,12,14). A significant increase in rate is noted in 2022 (n = 46) representing at least double the rate of five of the preceding six years. Clinical complaints are more predominant than non-clinical across a four year period (mean = 59% annual total) but no significant change in rate was noted. Rates of complaints are perceived to have increased in the previous five years by ECDs (n = 4). Repeat complainants are perceived to be common (n = 4). Workload regarding complaints is reported to be variable between services (n = 2, 0–4 hrs/week v. n = 2, 4–8 hrs/week). A clear appeals pathway is unavailable regarding clinical complaints across MHS (n = 4). A disparity between MHS around clinical complaints structures and recording practices between services is noted.

**Conclusion:**

Overall rates of complaints towards MHS have broadly increased in the last four years, with a significant increase in 2022. There appears to be a significant disparity in structures between both clinical and non-clinical complaints pathways and between individual MHS. Further research in this area and a standardised national framework for management of clinical complaints is needed.

